# Prevalence of psychological distress and mental disorders, and use of mental health services in the epidemiological catchment area of Montreal South-West

**DOI:** 10.1186/1471-244X-12-183

**Published:** 2012-10-30

**Authors:** Jean Caron, Marie-Josée Fleury, Michel Perreault, Anne Crocker, Jacques Tremblay, Michel Tousignant, Yan Kestens, Margaret Cargo, Mark Daniel

**Affiliations:** 1 Department of Psychiatry, McGill University, Douglas Mental Health University Institute Research Center, Montreal, Québec, Canada; 2 Centre for Research on Intervention on Suicide and Euthanasia, Université du Québec à Montréal, Montréal, Québec, Canada; 3 Département de Médecine sociale et préventive Université de Montréal, Centre de recherche du Centre Hospitalier de l’Université de Montréal, Montréal, Québec, Canada; 4 Social Epidemiology and Evaluation Research Group, Sansom Institute for Health Research, University of South Australia, Adelaide, Australia

## Abstract

**Background:**

This report presents the initial results of the first Epidemiological Catchment Area Study in mental health in Canada. Five neighbourhoods in the South-West sector of Montreal, with a population of 258,000, were under study. The objectives of the research program were: 1) to assess the prevalence and incidence of psychological distress, mental disorders, substance abuse, parasuicide, risky behaviour and quality of life; 2) to examine the links and interactions between individual determinants, neighbourhood ecology and mental health in each neighbourhood; 3) to identify the conditions facilitating the integration of individuals with mental health problems; 4) to analyse the impact of the social, economic and physical aspects of the neighbourhoods using a geographic information system. 5) to verify the adequacy of mental health services.

**Method:**

A longitudinal study in the form of a community survey was used, complemented by focused qualitative sub-studies. The longitudinal study included a randomly selected sample of 2,433 individuals between the ages of 15 and 65 in the first wave of data collection, and three other waves are projected. An overview of the methods is presented.

**Results:**

The prevalence of psychological distress, mental disorders and use of mental health services and their correlates are described for the first wave of data collection.

**Conclusion:**

Several vulnerable groups and risk factors related to socio-demographic variables have been identified such as: gender, age, marital status, income, immigration and language. These results can be used to improve treatment services, prevention of mental disorders, and mental health promotion.

## Background

This paper describes the objectives, the theoretical model and the methodology of a research program for the development of an epidemiologic catchment area in the South-West sector of Montreal. It also presents the results of the prevalence of psychological distress and mental disorders and the use of mental health services and their correlates for the first wave of data collection in this longitudinal study. More specifically, the influence of age and gender on the prevalences will be discussed, as well as the comorbidity of mental disorders by gender. Finally, vulnerable groups and socio-demographic risk factors for psychological distress and mental disorders will be presented.

This program can be classified among the “third generation of Psychiatric Epidemiology studies” [1], but it includes many innovative elements and methods. In the early eighties, the National Institute of Mental Health (USA) supported a program of epidemiological research based on community surveys, involving five U.S. sites of approximately 200,000 people [2]. The studies documented the changes in the incidence and prevalence of mental disorders and the use of mental health services among these populations over a period of several years.

In Canada, there had been no social and psychiatric epidemiologic catchment area prior to this program, although Canadians show high levels of mental distress. In a recent analysis of the Canadian Community Health Survey 1.2: (ESCC1.2), a cross-sectional study[3] shows a high prevalence of psychological distress and mental disorders in the general population. In addition to individual suffering, the minimum annual economic burden of psychological distress and mental disorders in Canada in 2003, including direct and indirect cost, is estimated at $51 billion [4].

The specific objectives of this mental health catchment area study were:

1) To assess the prevalence and incidence of psychological distress, mental disorders, substance abuse, pathological gaming, parasuicide and risky behaviour, as well as the quality of life of the population.

2) To examine the links and interactions between individual determinants, neighbourhood ecology and mental health in each neighbourhood.

3) To identify the conditions favouring the integration of individuals with mental health problems.

4) To understand the impact of the social, economic and physical aspects of neighbourhoods on mental health, using a geographic information system.

5) To verify the adequacy of mental health services.

### Theoretical model

**Figure 1:**
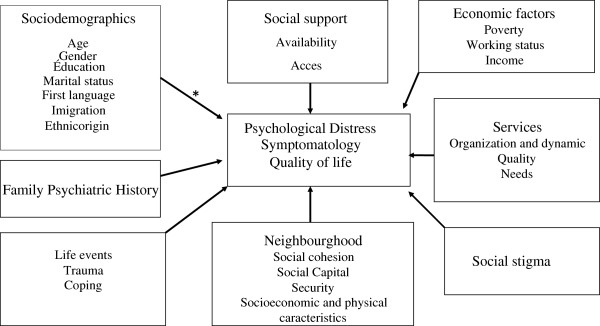
**Theoretical Model including variables related to Mental Health.** *The direction of the arrows does not indicate causality and several variables within each block are interrelated and probably interact with each other.

In order to achieve these objectives and to select instruments for measuring variables related to mental health and its determinants, we used the following theoretical model (Figure 1). This model was also used to determine the variables to be included into multivariate statistical analyses for identifying the various parameters related to mental health, mental disorders and service utilization.

The mental health of a population is the result of complex interactions among different parameters at the individual and population levels. Among risk factors, poverty remains the most critical parameter for developing psychological distress and psychiatric symptoms [3,5,6]. The physical environment and poor social conditions are producers of chronic stress and highly stressful life events [6,7]. However, social variables are the best protective factors of mental health. Among these variables, the perceived availability of social networks of support is the single best protective factor [8]. Social cohesion in communities also plays a significant role in maintaining healthy populations [9,10]. While social support refers to social network support, the concept of social cohesion refers to the degree of interaction, relationships and solidarity of social groups.

Many studies have shown that the physical characteristics of neighbourhoods have an impact on the mental health of its citizens [11,12]. A person’s conscious perception of his/her environment has also been identified as a key risk mediator “lying along an indirect cognitive path linking social structure to health.” [13] The concept of stress developed [14] provided a good basis for understanding the interaction between the biological and social dimensions of human adaptation. Research has shown that the accumulation of disruptive and stressful events has a negative impact on health [15] and that the ability to manage stress with adequate strategies leads to better adaptation [16,17].

Several models of adaptation based on the concept of stress have been proposed in the field of mental health. [17-19]. In general, they are based on the following premise: mental health and well-being are the result of a balance between the risk factors to which a population is exposed and the protective factors at its disposal. When symptoms of mental illness develop in a population, the quality of mental health services available to the community also plays an important role in maintaining this equilibrium. As a result, if formal and informal services are easily accessible and efficient, the duration of distress or symptoms will be shorter, thus reducing the prevalence of mental health problems in the population [20].

In addition, the social stigma associated with mental illness represents a major obstacle to recovery. It has negative impact on all stages of the disease: prognosis, treatment and outcome. Stigma is one of the most important factors impeding access to treatment, thus limiting the individual’s rehabilitation and ability to resume a normal and meaningful social life. Stigma adds to environmental stressors, promotes relapse and increases the burden of illness [21,22].

Figure 1 presents the theoretical model on which this program was based. This model includes a set of variables that are directly or indirectly related to mental health.

Quality of life is the positive mental health parameter, while psychological distress and a series of symptoms and behavioural measures (psychological disorders, substance dependence, gambling and crime) are considered as negative parameters. The various parameters associated with mental health, as described above, are part of the model. Socio-demographic indicators have also been added and they are associated with either a higher level of distress or, conversely, with a better quality of life. A number of studies show that people who develop symptoms have family histories that predispose them, although the interplay of genetic contribution and social interactions has not yet been clarified [23].

The particular strengths and originality of this research program include its combination of a quantitative longitudinal survey with quantitative and qualitative sub-studies of specific health determinants (services use and social stigma), as well as the integration of a unique geographic information system (GIS) for studying the neighbourhood social and ecological contexts [24]. The research model accounts for mental health services as one of the determinants of mental health, as suggested [25], and is able to compare neighbourhoods within its delimited study area.

## Method

### Setting

There were 269,720 people living in this zone. This area is divided into four boroughs: Saint-Henri/Pointe St-Charles (29,680), Lachine/Dorval (42,850), LaSalle (53,635) and Verdun (72,420).

### Sample

Our objective was to obtain a representative sample of the population between the ages of 15 and 65, with regard to geographical location, population density, and SES (based on the educational attainment structure of the territory). Of the 269,720 citizens, 198,585 were between the ages of 15 and 65. A random sample of 3,408 addresses was selected for recruitment using a list of addresses provided by the 2004 property evaluation role from the City of Montreal. To improve recruitment, we extended the original selected addresses to a range of 14 neighbouring addresses for door-to-door recruitment; the 3,408 original addresses thus resulted in a potential of 47,712 addresses.

The final sample of 2,433 participants represented approximately 600 participants in each borough: Saint-Henri/Pointe St-Charles (612), Lachine/Dorval (603), LaSalle (584) and Verdun (635), for a cooperation rate of 48.7%. This is superior to the median rates reported in epidemiological studies of populations conducted post year 2000 [26].

The study sample overrepresented women (61.6%) compared to the reference population (51.7%); men under the age of 45 were underrepresented. In order to obtain the precise prevalence of mental illness in the population, we weighted the data for sex and age. Table 1 presents sample characteristics before and after weighting.

**Table 1:** Socio-demographic characteristics of the sample (weighted)

	**Unweighted total**	**Weighted total**
	**(n = 2433)**	**(n = 2432.37)**
***Gender (%)***		
Female	1503	51.71
Male	930	48.29
***Age (mean, SD)***	41.39, 13.34	40.73,14.09
***Age (%)***		
15-24	292	16.12
25-34	525	20.66
35-44	574	20.84
45-54	546	20.92
55 +	496	21.46
***Marital status (%)***		
Single	886	37.95
Married	724	29.37
Separated	74	2.82
Common-law	384	15.86
Divorced	319	12.39
Widowed	42	1.61
***Education (%)***		
Less high school	372	15.99
High school	280	12.13
Post-high school	1780	71.88
***Immigrant (%)***		
No	1811	75.14
Yes	603	24.86
***Primary language (%)***		
English	528	20.59
French	1308	55.36
English + French	159	6.56
Neither English nor French	416	17.50
***Caucasian (%)***		
No	450	18.46
Yes	1958	81.54
***Dwelling owned by a household member (%)***		
No	1484	61.15
Yes	930	38.85
***Held a job in past 12 months (%)***		
No	545	21.41
Yes	1866	78.59
***Household size (mean, SD)***	2.50, 1.39	2.49,1.36
***Household income (mean, SD)***	$57,683, $49,718	$59,056, $49,851
***Personal income (mean, SD)***	$32,534, $31,200	$33,192, $33,151

The mean age was 40.73 (SD = 14.08) of whom 48% were men; 38% were single, 45% were married or in common law relationship, and 12% divorced or separated; 71% had a post-high school diploma; 79% were employed in the last 12 months; 25% were immigrants. French was the primary language spoken by 55% of the respondents, followed by 21% English; and 82% were Caucasian. The average personal income was CAN$ 31,192 (SD = $33,151) and the average family income CAN$ 59,056 (SD = $49,851); 33.4% of the participants were considered as having a low income according to the criteria of Statistics Canada.

### Instruments

This section presents all of the instruments used in the research program; however, psychometric properties are described only for instruments whose results are presented in this paper.

**Socio-demographic and economic data** were collected using the Canadian Community Health Survey questionnaire (CCHS 1.2) [27].

**Psychological distress** was measured using the **K-10** scale [28]. Its internal consistency yields an alpha coefficient of 0.93, its sensitivity level 0.45 and its level of specificity 0.92. This scale is used in the World Mental Health Survey (WMH2000), as well as in the in the CCHS 1.2. The psychological distress scores were dicotomized and the cut-off point for determining high psychological distress was 9 [3].

**Mental disorders** were identified with the CCHS 1.2 version of the **Composite International Diagnostic Interview**[27,29], including mood disorders (major depression, and mania), and some anxiety disorders: panic attacks, social phobia, and agoraphobia. The level of concordance between the CIDI and the ICD-10 is generally good (kappa ranging from 0.58 to 0.97). The level of sensitivity varies from 0.43 to 1, and the specificity ranges from 0.84 to 0.99, depending on the diagnosis.

**Alcohol and drug dependence** were assessed using a short form of the CIDI, (based on the DSM-III-R criteria). Previous versions of the CIDI have demonstrated reliability and validity [29,30].

The use of **mental health services questionnaire** was adapted from the CCHS 1.2 [26]. It measures the need for care and the type and frequency of service use (hospitals, local mental health community service centres, rehabilitation centres, private clinics, support groups and crisis services), as well as consultation with the following mental health professionals: psychiatrists, psychologists, general practitioners, case managers, toxicologists, nurses, psychotherapists, pharmacists and other health professionals.

Several other instruments consistent with our theoretical model were used. Impulsivity was measured using the **Barratt Impulsivity Scale**[31]. Self-reported aggressive behaviour was evaluated using the **Modified Overt Aggression Scale** (MOAS) [32]. Cognitive impairment was measured using the **Montreal Cognitive Assessment** tool [33]. Mental Health was measured with an adaptation of the **Satisfaction with Life Domains Scale**[34] and the Mental Health continuum short form [35]. **The Devaluation-Discrimination Scale**[22] was used to measure social stigma. Stress and stress management strategies were evaluated using the **CCHS 1.2 questionnaire**[26]. Social support was measured with the **Social Provisions Scale**[36].

Residents’ perception of their neighbourhood was measured using several instruments: **Sense of Community Index**[37], **Community Involvement Scale**[38], **Resident Disempowerment Scale**[39], **Sense of Collective Efficacy**[40], **Neighborhood Disorder Scale** and **Neighborhood Physical Conditions Scale**[39]. A geographic information system (GIS) was also used to assess the neighbourhood social and ecological contexts [24].

### Procedure

The project was approved by the Douglas Mental Health University Institute Ethics Committee, in accordance with the Canadian Tri-Council Guidelines. The interviewers contacted the residents who had agreed to participate in the study by phone within a week of recruitment, in order to schedule a face-to-face meeting either at the participant’s home or in an office designated for that purpose at the Douglas; however, most interviews were conducted at home. The face-to-face interview was conducted once the consent form was signed and lasted approximately 1.5 to 3 hours, depending on whether a mental disorder was detected.

### Statistical analyses

Descriptive statistics, including proportions, means and standard deviations (SD), were used to characterize the study population. Since all the outcome variables (high psychological distress, mental disorders, and substance dependences) are binary, we used a chi-square test to compare the prevalence of the outcome variables 1) between gender, and 2) across subgroups by age. A total of 5 age subgroups were compared. In addition to the chi-square test, a Cochran-Armitage trend test was used to determine if increasing or decreasing age influenced the prevalences.

We then conducted multivariable logistic regressions to explore how socio-demographic characteristics correlated with mental disorders. To avoid multiplicity problems due to performing many significance tests within one study, we restricted the outcomes to four disorder variables that are not alternative ways of measuring the same things: 1) any mood disorder; 2) any anxiety disorder; 3) any substance dependence; 4) any disorder or substance dependence. The following socio-demographic variables were included in each model: gender, age, marital status, household income, highest education, immigration status, primary language, and ethnicity. All analyses used SAS statistical software (version 9.2. Cary, NC).

## Results

### Prevalence of high psychological distress

Almost 4 out of 10 people interviewed, representing (38%) of the population of the catchment area, experienced symptoms of high psychological distress (Table 2). Women were more vulnerable than men (*X*^2^ = 7.11, p < 0.01). The 15-24 age group had the highest rate, while the 55 and older group had the lowest rate (*X*^2^ = 13.62, p < 0.01). The rates were especially high among single persons, and those who were widowed or divorced. This rate was significantly lower for married people and for people living in common-law (*X*^2^ = 47.95, p <0.001). The rate of psychological distress among separated people was equivalent to the average. The people who had a post-high school education were less affected, while those who had not completed a high school education were the most vulnerable (*X*^2^ = 24.59, p <0.001). Immigrants (*X*^2^ = 3.20, p = 0.074) and people whose primary language was neither English nor French (*X*^2^ = 6.89, p < 0.10) were less distressed than people whose primary language was either English or French. However, race status did not affect the level of psychological distress. (*X*^2^ = 0.88, p = 0.35).

**Table 2:** Prevalence of high psychological distress and selected past 12-month disorders by age and gender

**Symptoms**	**Gender**		**Age**						**Total**
	**M**	**F**	**15-24**	**25-34**	**35-44**	**45-54**	**55 +**	**Cochran-Armitage trend test (P-value)**	
	**n**	**n**	**n**	**n**	**n**	**n**	**n**		**n**
	**%**	**%**	**%**	**%**	**%**	**%**	**%**		**%**
**High psychological distress**	412.24	497.68	169.11	185.85	201.89	185.36	167.71	<0.001	909.92
	35.38	40.31*	43.99	37.59	40.08	36.94	32.53**		37.92
**Mood disorders**	87.74	141.64	31.20	48.78	57.28	58.21	33.91	0.61	229.38
	7.49	11.28**	7.96	9.76	11.30	11.44	6.52*		9.45
Depression	79.22	129.97	26.70	45.14	51.39	52.05	33.91	0.92	209.19
	7.05	10.73**	7.08	9.53	10.45	10.48	6.84		8.96
Mania	17.89	26.76	6.73	8.83	15.09	10.35	3.65	0.30	44.65
	1.54	2.15	1.72	1.81	3.00	2.05	0.71		1.86
**Anxiety disorders**	36.85	106.86	20.61	37.16	36.92	24.64	24.39	0.17	143.72
	3.14	8.50***	5.26	7.39	7.28	4.84	4.69		5.91
Panic	10.83	33.22	6.97	9.90	12.53	8.09	6.56	0.43	44.05
	0.93	2.69**	1.80	2.00	2.51	1.61	1.29		1.84
Social Phobia	21.04	59.04	12.25	23.63	19.31	12.48	12.42	0.11	80.08
	1.81	4.78***	3.18	4.75	3.84	2.49	2.43		3.34
Agoraphobia	8.87	20.18	2.71	7.04	7.91	5.21	6.18	0.78	29.06
	0.76	1.62	0.69	1.41	1.57	1.04	1.20		1.20
PTSD	4.17	13.59	3.77	5.65	4.14	3.72	0.48	0.047	17.76
	0.36	1.10*	0.97	1.14	0.84	0.74	0.10		0.74
**Substance dependence**	98.13	52.10	37.94	32.99	37.47	26.34	15.48	<0.001	150.23
	8.47	4.18***	9.71	6.63	7.42	5.22	3.04***		6.24
**Alcohol dependence**	65.35	31.15	24.60	16.55	25.69	19.32	10.35	<0.01	96.50
	5.71	2.52***	6.34	3.39	5.13	3.86	2.05*		4.05
**Drug dependence**	47.08	29.63	21.08	18.69	21.60	10.20	5.13	<0.001	76.71
	4.07	2.39*	5.45	3.76	4.28	2.03	1.01***		3.20
**All disorders**	176.74	229.21	69.96	90.38	92.20	90.74	62.68	0.02	405.95
	15.07	18.23*	17.84	17.98	18.19	17.83	12.06*		16.70

*** P < 0.001 **p < 0.01 * p < 0.05 from chi-square tests for comparing the sexes and across subgroups by age.

### Prevalence of mental disorders

The prevalence of mental disorders reached 16.7%; this percentage was slightly higher among younger of 15-24 years compared to those aged 55 and over. (Table 2). Mood disorders (9.5%) were more prevalent in women than in men and less prevalent in people over 55 than in younger people. The prevalence of anxiety disorders was 5.9% and women were overrepresented. Substance dependence was two times higher in men than in women while the prevalence stood at (6.2%).

### Prevalence of mood disorders

#### Depression

Major depression was the most common mental disorder (9%); the rate was higher in women than men (*X*^2^ = 10.09, p < 0.01), but the differences between the age groups were not significant (*X*^2^ = 7.20, p = 0.13). However, marital status did have a significant effect (*X*^2^ = 23.96, p < 0.001): depression rates were more than twice as high among participants who were either separated, widowed or single and almost double among those who were divorced compared to unmarried people and people living in common-law. Immigrants showed lower rates of depression than Canadian-born participants (*X*^2^ = 4.70, p <0.05). In addition, participants whose primary language was neither English nor French showed depression rates that were significantly lower than French or English Canadians (*X*^2^ = 12.88, p < 0.01). Education level (*X*^2^ = 4.33, p = 0.11) and race status (*X*^2^ = 2.10, p = 0.15) were not related to rates of depression.

#### Mania

The prevalence of mania was 1.9%; there was no socio-demographic correlate to this mental disorder other than the primary language (*X*^2^ = 25.16, p <0.001). Participants who described themselves as bilingual, fluent in both French and English, showed rates of mania that were three times higher than people whose primary language was either French or English. Participants whose primary language was neither English nor French showed the lowest rate of mania disorder.

### Prevalence of anxiety disorders

#### Social phobia

Social phobia stands out as the most prevalent disorder (3.3%) encountered in the population after depression. Women were more at risk than men to develop this disorder (*X*^2^ = 16.76, p <0.001). Marital status had a significant impact on the prevalence of this anxiety disorder (*X*^2^ = 11.78, p < 0.05): participants who were widowed or living in common-law relationships had the highest rates of social phobia. In contrast, those who were married showed the lowest rate, followed by divorced, single or separated men and women. Immigrants scored the lowest rate for social phobia compared to non-immigrants (3.8%) (*X*^2^ = 5.70, p < 0.05). Age and other socio-demographic correlates were unrelated to social phobia.

#### Panic

The prevalence of panic disorder was 1.84%; women were affected three times as much as men (*X*^2^ = 10.39, p = <0.01). In addition, this disorder was three to eight times more prevalent among people who were widowed compared to those who were divorced, married, separated or in common-law relationships. Individuals who were single were more prone to panic attacks (2.7%) (*X*^2^ = 18.22, p = <0.01). Age, education and other demographic variables were unrelated to this disorder.

#### Agoraphobia

Agoraphobia was detected in 1.2% of the population. Women were more likely to be affected than men (0.08%) (*X*^2^ = 3.81, p < 0.05). A trend was detected with respect to marital status (*X*^2^ = 10.36, p = 0.06): people who were either divorced or single appeared to be more vulnerable. Other socio-demographic correlates were unrelated to agoraphobia.

### Prevalence of substance dependence

#### Alcohol dependence

The prevalence of alcohol dependence was 4.1% in the total population. Gender (*X*^2^ = 15.36, p <0.001), age (*X*^2^ = 12.33, p < 0.05), marital status (*X*^2^ = 45.87, p <0.001), education level (*X*^2^ = 8.01, p = 0.018), immigration status (*X*^2^ = 9.57, p < 0.01) and primary language (*X*^2^ = 12.54, p < 0.01) were all associated with alcohol dependence. The prevalence of alcohol dependence was twice as high for men as for women; the highest prevalence was found in the 15-24 age group, followed by the 35-44 group, the 25-34 group, and the 45-54 group which was closer to average. Those aged 55 and over showed the lowest rate. Individuals who were single were the most vulnerable, while those who were married or widowed had the lowest rate; those who were divorced, separated or living in common-law relationships were closer to the average. People who had achieved only a high school diploma were more vulnerable and the prevalence of alcohol dependence among those with a post-high school diploma was lower. Participants who had not achieved a high school degree were closer to the average. The rate of dependence was more than twice as high among non-immigrant Canadians compared to immigrants. Finally, people whose primary language was neither English nor French showed a dependence rate significantly lower compared to French or English Canadians. Participants who described themselves as bilingual, fluent in both French and English, showed a rate of alcohol dependence that was almost twice as high as those whose primary language was French.

#### Drug dependence

The prevalence of drug dependence was 3.2% in the general population. Gender (*X*^2^ = 5.27, p < 0.05), age (*X*^2^ = 18.70, p = 0.001), marital status (= 53.43, p <0.001), education level (*X*^2^ = 8.56, p <0.05), immigration (*X*^2^ = 12.35, p = 0.001) and first language (= 12.82, p <0.01) were again all correlated with drug dependence. The prevalence of drug dependence was nearly twice as high for men as it was for women, and this prevalence decreased linearly with increasing age. Single persons constituted the most vulnerable group followed by those who were separated, living in common-law relationships or divorced. People who were widowed showed a much lower rate of drug dependence; no dependence was detected among married men and women. Participants who had completed a post-high school diploma were the least vulnerable to drug dependence, and this prevalence was about half that of those with only a high school diploma or no diploma. The dependence rate was four times lower amongst immigrants compared to Canadian-born participants. Finally, participants whose primary language was neither English nor French showed dependence rates significantly lower than French or English Canadians. Participants who described themselves as bilingual, speaking both French and English, showed a drug dependence rate that was twice as high compared to those whose primary language was either French or English.

### Comorbidity

The average number of assessed disorders was 1.47 (SD = 0.81) among those presenting at least one disorder. There was no significant difference between men (X = 1.44, SD = 0.88) and women (X = 1.50, SD = 0.78) for the number of disorders. Among the participants who had an affective disorder, 27.6% presented at least one anxiety disorder and 19.8% had at least one substance dependence (either alcohol dependence or drug dependence). Among those suffering from an anxiety disorder, 44.6% presented at least one affective disorder and 17.8% had at least one substance dependence. Among those coping with substance dependence, 29.43% presented at least one affective disorder and 16.65% suffered from at least one anxiety disorder.

A significant difference in the comorbidity pattern between men and women was noted. On the one hand, men presenting affective disorders were more likely to have a co-occurring substance dependence (29.5%) compared to women (14%)(X^2^ = 7.92, p < 0.01). On the other hand, women with affective disorders showed increased likelihod of comorbid disorders related to anxiety disorders (32.2%) compared with men (20.2%) (X^2^ = 3.91, p < 0.05). In addition, men with anxiety disorders had a tendency to have more comorbid substance disorders (28.0%) than did their female counterparts (14.2%) (X^2^ = 3.57, p < 0.06). However, the co-occurring disorder patterns related to affective disorders were quite similar across genders. Women coping with substance dependence had to deal with a higher rate of comorbidities (49.4%) than men (28.8%)(X^2^ = 6.30, p < 0.05): 37.4% of women also present affective disorders, as compared to 25.2% of men, and 28.2% anxiety disorders, as compared to 10.5% of men.

### Socio-demographic correlates of mental disorders

Men had a lower risk of suffering from affective (OR = 0.62) or anxiety (OR = 0.39) disorders than women, but the substance dependence rate was twice as high for men compared to women (OR = 2.11). For all disorders there was no significant difference between genders. (Table 3).

**Table 3:** Multivariable logistic model for past 12-month disorders

	**Any mood disorder**		**Any anxiety disorder**		**Any substance dependence**		**Any disorder**	
	**(n = 229.38)**		**(n = 143.72)**		**(n = 150.23)**		**(n = 405.95)**	
	**OR**	**95%CI**	**OR**	**95%CI**	**OR**	**95%CI**	**OR**	**95%CI**
***Gender***								
Women (ref)	1.00	…	1.00	…	1.00	…	1.00	…
Men	0.62	0.45-0.84 ^*^	0.39	0.26-0.58 ^*^	2.11	1.43-3.11 ^*^	0.80	0.63-1.02
***Age***								
15-24	1.21	0.64-2.31	0.98	0.45-2.12	2.16	1.01-4.66 ^*^	1.39	0.86-2.26
25-34	2.05	1.18-3.53 ^*^	1.78	0.93-3.40	2.39	1.14-5.02 ^*^	2.11	1.37-3.23 ^*^
35-44	2.39	1.42-4.01 ^*^	2.13	1.14-4.00 ^*^	3.01	1.49-6.10 ^*^	2.31	1.53-3.49 ^*^
45-54	2.48	1.53-4.04 ^*^	1.28	0.66-2.47	2.21	1.10-4.45 ^*^	2.19	1.48-3.25 ^*^
55 + (ref)	1.00	…	1.00	…	1.00	…	1.00	…
***Marital status***								
Single	1.31	0.84-2.03	1.77	0.99-3.18	6.02	2.66-13.60 ^*^	1.73	1.21-2.48 ^*^
Married (ref)	1.00	…	1.00	…	1.00	…	1.00	…
Separated	1.29	0.56-2.96	1.15	0.34-3.91	3.98	1.09-14.57 ^*^	1.75	0.89-3.44
Common-law	0.90	0.53-1.56	2.36	1.25-4.48 ^*^	3.40	1.39-8.37 ^*^	1.42	0.94-2.14
Divorced	1.19	0.70-2.04	0.92	0.41-2.05	3.99	1.57-10.14 ^*^	1.18	0.75-1.86
Widowed	2.47	0.91-6.73	3.43	1.08-10.86^*^	1.43	0.09-23.99	2.89	1.24-6.76 ^*^
***Income***								
0-19,000	4.14	2.51-6.83^*^	4.60	2.40-8.83 ^*^	3.07	1.65-5.69^*^	4.12	2.79-6.10 ^*^
20,000-34,000	2.05	1.20-3.50 ^*^	3.44	1.80-6.61 ^*^	2.40	1.28-4.49 ^*^	2.36	1.58-3.51 ^*^
35,000-69,000	2.13	1.37-3.32 ^*^	2.25	1.25-4.06^*^	1.66	0.95-2.92	1.78	1.27-2.51 ^*^
70,000+ (ref)	1.00	…	1.00	…	1.00	…	1.00	…
***Education***								
Less than high school	0.94	0.61-1.45	1.09	0.66-1.81	1.06	0.64-1.76	1.04	0.74-1.46
High school	1.26	0.80-1.98	0.80	0.42-1.54	1.46	0.85-2.49	1.21	0.84-1.75
Post-high school (ref)	1.00	…	1.00	…	1.00	…	1.00	…
***Immigrant***								
No (ref)	1.00	…	1.00	…	1.00	…	1.00	…
Yes	0.93	0.53-1.64	1.11	0.56-2.20	0.31	0.13-0.74 ^*^	0.77	0.49-1.23
***Primary language***								
English	0.98	0.67-1.43	1.03	0.64-1.65	1.27	0.79-2.03	1.15	0.85-1.55
French (ref)	1.00	…	1.00	…	1.00	…	1.00	…
English + French	1.10	0.62-1.95	1.05	0.52-2.14	1.18	0.62-2.27	1.00	0.63-1.60
Neither EN nor FR	0.43	0.22-0.84 ^*^	0.40	0.17-0.95 ^*^	0.70	0.26-1.90	0.45	0.26-0.79 ^*^
***Caucasian***								
No (ref)	1.00	…	1.00	…	1.00	…	1.00	…
Yes	1.01	0.58-1.76	1.09	0.55-2.16	0.81	0.38-1.69	1.16	0.74-1.82

^*^ P < .05 from the two-sided wald chi-square test based on multivariable logistic model with adjustment for all the other socio-demographic variable.

Age was systematically related to mental disorders. Men and women 55 years and over showed the lowest rates of any type of measured mental disorder. Every age group, that is, 25-35 (OR = 2.05), 34-45 (OR = 2.39), and 45-55 (OR = 2.48), showed a risk factor for mental disorder that was twice as high for an affective disorder or for any other disorder compared to those aged 55 and over; however, those 34-45 presented a higher risk for anxiety disorders (OR = 2.13). All the age groups had a higher risk for substance dependence (twice as high) compared to the 55 and over group, with odds ratios varying between 2.16 and 3.01.

Marital status was not related to affective disorders; however, people living in common-law relationships (OR = 2.36) or who were widowed (OR = 3.43) showed an increased risk for anxiety disorders. All participants whose income was lower than $70,000 were at increased risk for each disorder; the risk increased systematically as income decreased. For example, for any disorder, the OR was 4.12 for those whose income was less than $19,000 and decreased to 1.78 for those whose income was between $35,000 and $69,000. The level of education was unrelated to any category of disorders.

Immigrants showed a lower risk (OR = 0.31) for drug dependence than non-immigrants. In addition, participants whose primary language was neither French nor English were less at risk than Francophones or Anglophones for developing substance dependence, for affective (OR = 0.43) and anxiety disorders (OR = 0.40), or for any disorders (OR = 0.45), with the exception of substance dependence. Race was not related to mental disorders.

### Use of mental health care services

Among the 406 participants who experienced at least one episode of mental illness, 212 (52%) reported using mental healthcare services at least once in their lifetime. These 212 participants had been affected mainly by major episodes of depression (N = 129; 61%).

## Discussion

A high rate of psychological distress was found in the population of the catchment area (38%), almost twice that reported in the Canadian Community Mental Health Survey: 1.2 (21%). The rate of mental disorders was also higher in the sample population, 17% compared to 11% in the Canadian population [3]. This could be explained by the lower economic level of the population of this catchment area, where 33.4% of the sample population interviewed reported belonging to the lower-income group compared to 19% for the rest of the Canadian population. Participants earning less than $19,000 annually were 4.3 times more at risk of having any type of mental disorder compared to those whose income was over $70,000. The CCHS 1.2 showed a significantly higher rate of psychological distress (50% higher) as well as a higher rate of mental disorders (35% higher) in less fortunate populations compared to populations enjoying a higher income [3,41]. The survey confirmed what most epidemiological studies conducted around the world have concluded to date: people belonging to the lowest socio-economic level of our society are the most vulnerable to psychological distress [42-44]. There is considerable evidence that underprivileged groups are affected by chronic stress and more negative life events [11,44]; their social networks are not as strong and reliable as higher income groups [9,45].

In addition, comorbidity of mental disorders was high among subjects suffering from an affective disorder: approximately 40% of these people were coping with another disorder. Women had more co-occurring mental disorders with affective and depressive disorders compared to men who showed higher rates of substance dependence and other types of disorders. These comorbidity patterns are quite similar to those described in the Australian population [46].

Women in the catchment area reported higher rates of psychological distress, depression and anxiety disorders than men, and the results were consistent with most epidemiological studies [47-51]. Men showed a higher rate of substance-related disorders, as reported in previous studies [48,49,52-54].

We also found a correlation between (older) age and lower levels of psychological distress, lower rates of affective disorders, anxiety disorders and substance dependence. These results are also consistent with the literature [48,50,55,56]. Stephens, Dulberg & Joubert [57] reported in a cross-Canadian health survey that the probability of having a better sense of coherence significantly increased with age and that self-esteem and a feeling of happiness reached a peak between the ages of 40 and 59.

Consistently across specific disorders and for psychological distress, married people were at lower risk. In crude associations, single people showed higher rates of alcohol and drug dependence, agoraphobia and psychological distress. People who were separated experienced higher rates of depression, while divorced people showed higher rates of psychological distress and agoraphobia. Those who were widowed were at increased risk for psychological distress, panic disorders, depression and social phobia, and participants living in common-law showed higher rates of social phobia. When marital status was included in regression analysis for controlling other variables in broader categories of disorders, most of the relationships identified with crude association disappeared. Those widowed or living in common-law relationships were at higher risk for anxiety disorders or any disorders, while single, divorced, separated and common-law participants were at increased risk for substance dependence. It is well known that being single, separated or divorced decreases social support and is also associated with lower income, the latter being one of the strongest predictors of psychological distress and mental disorders in this study, as well as in many others (see Caron and Liu, [41]).

Most studies generally report that being married protects couples from psychological distress and other disorders compared to people who are either widowed, divorced, separated, or single [48,52]. However, some reports have indicated that this relationship between marital status and distress was limited to younger couples (< 44 years) [50]. Jorm *et al.*[55] reported that there was no significant correlation between marital status and the level of psychological distress, while Bahadur and Hauff [51] reported that the absence of a partner increased the level of distress for women only.

Education was associated with specific psychological distresses as well as drug and alcohol dependence. Post-high school education was negatively associated with psychological distress and this result is consistent with previous studies [55,56]. Moreover, a post-high school education also lowered the risk of developing drug dependence, but a high school education increased the risk of alcohol dependence. However when education was controlled for other variables in regression analysis, it lost its relationship in any category of disorders because it was strongly associated with income.

In crude association, immigration and first language other than French and English were systematically related to lower psychological distress and specific disorders. When controlled for other socio-demographic variables in regression, immigration lowered the risks of developing substance dependence only, and participants whose primary language was neither French nor English had lower risks of developing affective and anxiety disorders and substance dependence. This would suggest that language and immigration have a distinct relation to mental health. People with a first language other than French or English (official languages in Canada) probably belong to a cultural community, and come from close-knit families, some of whom may have immigrated to Canada many years ago, with whom they share protective factors associated with their cultural groups. These results are consistent with those reported in the Canadian Community Health survey 1.2, in which immigrants showed systematically lower psychological distress and mental disorders [3,41]. In the Ethnic Diversity Survey [58], immigrants reported a strong sense of belonging to their ethnic or cultural group compared to Canadian-born citizens, and regardless of their time of arrival in Canada, were also more likely to be involved in ethnic or immigrant associations compared to Canadian-born individuals. Sense of belonging was also identified in several studies as a predictor of lower psychological distress [10,44,47]. Lower psychological distress and mental disorders among immigrants may reflect the current immigration policy, which excludes immigrants with potential chronic diseases such as mental illness for admission to Canada.

Of the participants diagnosed with mental disorders, 52% used health care services provided in the catchment area. Most studies find that approximately a third of people suffering from mental disorders used healthcare services to help them with their mental disorders [59,60]. The proximity of a psychiatric hospital in the catchment area may account for the overall greater healthcare service use, and individuals with low income have easy access to the public healthcare system (general practitioners and medication).

A profile of services utilisation and predictors of service use related to socio-demographics and to social and neighbourhood variables have been identified for this catchment area, using the geographic information system, and have been published elsewhere [61-64].

### Limits and strengths of the study, and future direction of the program

This study has some limitations. Although the sample size and design were representative of the catchment area populations, the data of the first cycle presented here are cross-sectional and do not allow us to infer causal relationships between the correlates and identified mental disorders. However, as this program is prospective and designed to have at least four waves of data collection, causal inferences as to changes in mental health status and service use will become possible for future cycles. This type of prospective longitudinal study provides information on the effects of the variation in the determinants of mental health across time and on the mental health of the population, and contributes to strengthening causal hypotheses. In this paper, only socio-demographic variables have been correlated to mental disorders. However, other papers are in the works using multivariate models to assess the effects of a number of personal variables (life events and coping strategies), social variables (social support) and other variables related to neighbourhood (residents’ perception and objective data from the geographic information system) on mental illness and mental health.

This program is innovative in several ways. It is the first epidemiological catchment area to include measures of positive mental health, such as psychological well-being and quality of life. It also includes measures of impulsivity and criminal behaviour that can be linked to mental disorders. In addition, this is the only catchment area that uses measurements of the residents’ perception of various aspects of their neighbourhood and that employs a geographic information system to assess the effects of the social and built environment. Longitudinal studies using only quantitative methods do not enable us to understand the dynamics and functioning of the determinants of mental health. In order to understand these aspects, two qualitative studies have been conducted on subsamples of our cohort to better grasp the process of social stigma and services utilization in our catchment area. These innovations may lead the way to a fourth generation of studies in psychiatric and social epidemiology.

## Conclusion

This study has established the prevalence of psychological distress and the major groups of mental disorders by age and gender, in the epidemiological catchment area of Montreal South-West for the first wave of data collection. In addition, several vulnerable groups and risk factors related to socio-demographic variables have been identified such as: gender, age, marital status, income, immigration and language. Of these, note that low income is strongly associated with the prevalence of high psychological distress and mental disorders and that this variable is likely responsible for higher rates of prevalence in this area of Montreal than in Canada as a whole. As the research program was developed with all stakeholders of mental health services in this area and as they are involved in the knowledge transfer committee of the program, these data will certainly be useful for improving treatment services, prevention of mental disorders and mental health promotion.

## Competing interests

The authors declare that they have no competing interests.

## Authors’ contributions

The conception and the design of the Epidemiological Catchment Area needed a multidisciplinary team with the contribution of the expertise of many researchers. J C director of the CIHR team has developed the general design, MP, JT, JC were leading the aspects of mental disorders and co morbidity. M-JF and MP were responsible for developing the part on services utilisation, AC was responsible for criminal behaviour and impulsivity, MT was leading the cultural aspect of the project, MC was in charge of all measures on perception of the neighbourhoods and YK and MD have developed the geographic information system. JC and MT were responsible for data collection. Analyses of data were realized by JC but each author has been involved in interpretation according to their speciality. The article was mainly drafted by JC but each author has done a significant contribution when revising it. Finally all authors have approved the final version.

## Pre-publication history

The pre-publication history for this paper can be accessed here:

http://www.biomedcentral.com/1471-244X/12/183/prepub
